# Mutation at Different Sites of Metal Transporter Gene *OsNramp5* Affects Cd Accumulation and Related Agronomic Traits in Rice (*Oryza* sativa L.)

**DOI:** 10.3389/fpls.2019.01081

**Published:** 2019-09-11

**Authors:** Tiankang Wang, Yixing Li, Yuefeng Fu, Hongjun Xie, Shufeng Song, Mudan Qiu, Jiong Wen, Muwen Chen, Ge Chen, Yan Tian, Chengxia Li, Dingyang Yuan, Jianlong Wang, Li Li

**Affiliations:** ^1^College of Agronomy, Hunan Agricultural University, Changsha, China; ^2^State Key Laboratory of Hybrid Rice, Hunan Hybrid Rice Research Center, Changsha, China; ^3^Yueyang Agricultural Science Research Institute, Yueyang, China; ^4^Hunan Rice Research Institute, Changsha, China

**Keywords:** rice, Cd, *OsNramp5*, yield, quality

## Abstract

*OsNramp5* is a key gene involved in the control of the uptake of Cd, Mn, and other metal ions by rice root cells. The functional deficiency of this gene can significantly reduce the accumulation of Cd in rice grains, but the effects of its mutation on agronomic traits such as yield and quality have not been investigated comprehensively yet. In the present study, three Huanghuazhan-based *OsNramp5* mutants [*LCH1* (*Low Cadmium Huanghuazhan 1*), *LCH2* (*Low Cadmium Huanghuazhan 2*), and *LCH3* (*Low Cadmium Huanghuazhan 3*)] were obtained using clustered regularly interspaced short palindromic repeats/CRISPR-associated protein 9 (CRISPR/Cas9) technology. The mutation-type analysis showed that *LCH1*, *LCH2*, and *LCH3* encoded defective OsNramp5 protein sequences containing only 76aa, 176aa, and 266aa, respectively. The determination of metal content and the statistics of related agronomic traits revealed that the functionally deficient *OsNramp5* not only significantly reduced the accumulation of Cd in the grains of the mutants but also affected rice yield and quality. However, with the decrease of *OsNramp5* mutation degree, its effects on chlorenchyma Mn accumulation, yield, and quality were also diminished. Additionally, we also found that the increase in the concentration of Mn in the soil restored the phenotype of the declined yield and quality due to the functional deficiency of *OsNramp5*. Our findings provide novel insights into and new materials for breeding rice varieties with low Cd accumulation and excellent agronomic traits under severe Cd pollution environment.

## Introduction

Rice is one of the most important food crops in the world. At present, it is the staple food of more than half the world’s population (Wang et al., 2013). Cd is carcinogenic to humans and has been classified as a Class I carcinogen by the International Agency for Research on Cancer (Broerse et al., 2012; Alvarez-Olmedo et al., 2017). Cd is mainly absorbed from the soil through the roots and reaches the vascular bundle through the two plant transport pathways—the symplast and the apoplast—by which it is then transported to the stems and leaves and later migrates to the grains, finally accumulating in the human body through the food chain, thereby causing serious harm to human health (Yao et al., 2003; Seregin and Kozhevnikova, 2008; Yoneyama et al., 2015).

Currently, the following four methods are mainly used to reduce the Cd content in rice grains. The first is the removal of Cd in the soil or the change of the existing form of soil Cd by physical or chemical methods (Seregin and Kozhevnikova, 2008; Zhang Q et al., 2017; Mortensen et al., 2018). The second is inhibition of the formation of exchangeable Cd in the soil through cultivation measures such as water and fertilizer management (Cattani et al., 2008). The third is screening out low-Cd-accumulating cultivars from existing rice varieties (Zhang Y et al., 2017; Tang et al., 2018). The fourth is to reduce the content of Cd in the soil through phytoremediation (Shao et al., 2017; Acosta et al., 2018). These four approaches have considerable limitations in practice. For example, the restoration of Cd-contaminated soil is exceedingly costly and can lead to secondary soil pollution (Zaborowska et al., 2012; Ren et al., 2018). Different cultivation measures are used to address this issue, based on the specific soil types or rice varieties, but their effect is substantially affected by environmental conditions (Li et al., 2015; Yang et al., 2017). Screening for low-Cd-accumulating cultivars requires multi-spot verification over many years, and many screened varieties still have high accumulation of Cd in heavy Cd-contaminated fields (Sun et al., 2016; Gong et al., 2016). Phytoremediation is difficult to meet the need of rapid remediation of polluted environment due to its long remediation cycle (Li et al., 2017). With the rapid development of molecular biology, the major gene *OsNramp5*, involved in Cd uptake in rice, has been cloned, which enables a significant reduction in rice Cd accumulation through more economical, rapid, and efficient methods (Ishikawa et al., 2012).

*OsNramp5* is the main transporter in rice root cells involved in the absorption of external Cd, Mn, Fe, and Zn, which is also responsible for the transport of these ions from the root to the above-ground parts (Ishikawa et al., 2012; Ishimaru et al., 2012; Ishimaru et al., 2012; Yang et al., 2014). Therefore, the measures to reduce the Cd content in rice by blocking the absorption of soil Cd by the rice root cells *via* a mutation of the *OsNramp5* gene have begun to attract scientific attention in recent years. Studies have shown that three *OsNramp5* mutants *lcd-kmt1∼3* can be obtained by Koshihikari mutagenesis using carbon ion beam irradiation (Sasaki et al., 2012). Among them, the ninth exon of *OsNramp5* is deleted by 1 bp in *lcd-kmt1*. The 433bp transposon is inserted into the posterior part of the 10th exon of *OsNramp5* in *lcd-kmt2*, and the *OsNramp5* and 227-kbp at its two wings are deleted in *lcd-kmt3*. The Cd content of the *lcd-kmt1∼3* grains decreased to 0.02 mg/kg [1.86 mg/kg in wild type (WT)], and *lcd-kmt1* and *lcd-kmt2*, compared with the WT, exhibited no significant difference in the grain yield, whereas the *lcd-kmt3* grain yield seriously declined (Sasaki et al., 2012). The random insertion of Zhonghua 11 into the 12th intron of *OsNramp5* using T-DNA resulted in a decrease of the Cd content in the grains to approximately 0.01 mg/kg (about 0.16 mg/kg in the WT), whereas the grain yield was reduced to only 11% of the WT (Ishikawa et al., 2012). Clustered regularly interspaced short palindromic repeats/CRISPR-associated protein 9 (CRISPR/Cas9) technology was used in an earlier study to edit the PS1 and PS2 targets of the ninth exon of Huazhan *OsNramp5*, the results of which revealed that after the treatment with multiple soil Cd concentrations, the Cd contents in these two mutants were less than 0.05 mg/kg (0.33–2.90 mg/kg in the WT), whereas the grain yield did not differ significantly from that of the WT (Tang et al., 2017). Therefore, the above *OsNramp5* mutants obtained by different methods significantly reduce the Cd content in rice grains but exert different effects on rice grain yield, which becomes an obstacle in the process of using *OsNramp5* mutation that needs to be urgently overcome to create low-Cd-accumulating rice varieties.

In this study, the high-quality *Oryza sativa* subsp. *indica* Huanghuazhan was considered the acceptor, and the CRISPR/Cas9 technology was utilized to obtain the frameshift mutants *LCH1*, *LCH2*, and *LCH3* in the second, sixth, and ninth exon regions of *OsNramp5*, correspondingly. These three mutants exerted different effects on the natural resistance-associated macrophage protein (Nramp) and transmembrane domains of OsNramp5, encoding a defective OsNramp5 protein sequence containing only 76aa, 176aa, and 266aa, respectively. The metal accumulation, yield, and quality traits, as well as the expression of the Mn transporter gene, were analyzed. Our results showed that the functional deficiency of *OsNramp5* not only significantly reduced the accumulation of Cd in the grains of the mutants but also affected the yield, the head rice rate, and the chalkiness of the individual plant. We found that selecting the appropriate *OsNramp5* mutation position alleviated the effect of *OsNramp5* mutation on rice yield and quality.

## Materials and Methods

### Construction of the CRISPR/Cas9 Editing Vector

The CRISPR/Cas9 editing vectors, single-guide RNA (sgRNA) expression vector (*pYLgRNA-U3*), and the plant expression vector (*pYLCRISPR/Cas9-MH*) were provided by Ma et al. (2015). First, target site 1 (TS1), target site 2 (TS2), and target site 3 (TS3) of *OsNramp5* and the corresponding reverse complement sequences formed pairs of primers (GGCA and AAAC linker were added to the 5′ terminals of the sense and antisense primers, respectively), and three double-stranded target sequences [double-stranded target sequence 1 (DS-TS1), double-stranded target sequence 2 (DS-TS2), and double-stranded target sequence 3 (DS-TS3)] were obtained after annealing. Subsequently, DS-TS1, DS-TS2, and DS-TS3 were inserted into pYLgRNA-U3, followed by polymerase chain reaction (PCR) amplification to obtain three expression cassettes: *U3-DS-TS1-gRNA*, *U3-DS-TS2-gRNA*, and *U3-DS-TS3-gRNA*. Further, the three expression cassettes were separately inserted into *pYLCRISPR/Cas9-MH*, so as to obtain the CRISPR/Cas9 knockout vectors at TS1, TS2, and TS3.

### Callus Induction in Rice

Full, pest-free rice seeds were screened out, and the seed shell was removed with the roughing machine. Seeds were sterilized by soaking in 75% ethanol for 1 min and then transferred and incubated in 10% sodium hypochlorite solution containing Tween 80 for 15 min. This procedure was repeated three times. Seeds were then washed with sterile deionized water five times, and moisture was blown off on the ultra-clean workbench. After 1 h, seeds were transferred to the induction medium and cultured at 28°C in a dark room for 8 h. After about 10 days, when the callus of the seeds becomes pale yellow, the callus can be separated from the endosperm and transferred to a new induction medium. A week later, the infection of *Agrobacterium tumefaciens* could be carried out.

### Transformation of Huanghuazhan Callus With CRISPR/Cas9 Editing Vectors

After the callus was grown, the CRISPR/Cas9 editing vector was used to transform the callus by the Hiei and Komari, 2008 method and was then cultured in co-culture medium in a dark room at 28°C for 2.5 days. Subsequently, the material was transferred to the screening medium (containing 50 mg/L of hygromycin for screening transgenic callus) and cultured in a dark room at 28°C for 10 days. These steps were repeated two times in 10 days. Then the new callus was transferred to differentiation medium, cultured at 28°C, and exposed to light for 16 h (dark for 8 h) per day. The differentiation medium was replaced every 10 days. When the green rootless seedlings were grown, the seedlings were transferred to the rooting medium. When there grew a large amount of roots and the top of the seedlings capped against the lid of the rooting box, the cap was removed to select seedlings. After 10 days, the seedlings were moved to the field.

### Acquisition and Detection of Transgenic Plants

After seedlings were obtained by tissue culture, the genomic DNA was extracted from the leaves. Then, according to the vector sequence, pairs of primers (SP1/SP2) were designed to PCR-amplify DNA for each plant. The target band length was approximately 500-bp; the positive plants were screened. After that, a pair of detection primers was separately designed for two ends of the three target sites of the *OsNramp5* sequence, and the positive plant DNA was subjected to PCR amplification and afterwards sequenced by the Sanger et al. (1977) method. By aligning the sequence with the WT sequence, if mutations occur at the target site, the plant was identified as *OsNramp5* mutant. The primer sequences are listed in **Supplementary Data 2**, **Table S2**. The mutations of the three target sites are shown in **Figure 2**.

### Whole-Genome Resequencing

The *LCH1*, *LCH2*, *LCH3*, and WT seedlings were cultured in water, and young leaves were collected from each group during the tillering period. DNA was extracted, and the samples were sent to Mega Genomics (Beijing) Co., Ltd. The DNA was broken into small fragments of 200-500 bp by ultrasound, and *LCH1*, *LCH2*, *LCH3*, and WT were resequenced using a second-generation sequencer, Illumina NovaSeq 6000. The sequencing depth was 50×, and the reference genome was *O. sativa* L. spp. *japonica* (Margulies et al., 2005).

### Pot Experiment

The potted basal soil was the typical paddy soil; after air drying, grinding, and filtering processes with a 20-mesh, by applying CdCl_2_ and MnSO_4_, the soil Cd concentrations were set at 1 and 5 mg/kg, the Mn concentration was set to 350 mg/kg. Meanwhile, another group was treated with Cd of concentration 1 mg/kg and Mn of concentration 700 mg/kg. Each pot was filled with 10 kg of soil and pre-cultured with deionized water for 25 days. *LCH1*, *LCH2*, *LCH3*, and control seeds were bred in Cd-free soil and transplanted to a pot with gradient Cd concentration after 28 days, one plant per well and three plants per pot. Cultivation management was consistent with that for local high-yield paddy fields.

### Determination of Rice Yield Data

*LCH1*, *LCH2*, *LCH3*, and control grains and straw samples, treated with Cd concentrations of 1 and 5 mg/kg, were harvested and dried for testing. The indicators to be analyzed included yield per plant, straw yield, 1,000 grain weight, grain number per ear, seed-setting rate, and number of tillers (Li et al., 2011).

### Determination of Rice Quality

The methods for determining the brown rice rate, milled rice rate, and head rice rate were performed using the NY/T 593-2013 standard published by the Ministry of Agriculture of China (http://www.zbgb.org/27/StandardDetail1476335.htm).

Determination of grain length and aspect ratio of grains was as follows: 10 randomly chosen grains from the whole milled rice sample were arranged along the longitudinal direction of the vernier caliper; then the grain length was measured and calculated, including the grain width; the ratio of the grain length to the width was the aspect ratio of grains.

Determination of chalky grain rate and chalkiness degree was as follows: 100 head grains were randomly taken out, and grains containing chalkiness (including white heart, belly white, and back white) were picked out on the rice chalkiness observer (JC08-SDE-A). The observation was repeated once, and the percentage of grains containing chalkiness to the total number of polished rice samples was the chalky grain rate. Twenty grains containing chalkiness were randomly taken out, and the percentage of chalky area to the whole grain area was visually measured on the rice chalkiness observer (JC08-SDE-A). The average value of the chalky area was determined and repeated once. The average of the results was the chalkiness size. The product of the chalkiness size and the chalky grain rate was the chalkiness degree.

Determination of amylose content and gel consistency was as follows: the kernels were ground to a powder and measured according to the method previously described by Tan et al. (1999).

The protein content was determined using an XDS Near Infrared Rapid Content Analyzer (FOSS) according to the method previously described by Li et al. (2014).

### Determination of Metal Content

Grains and straw samples were cooked with a composition of nitric acid–perchloric acid (4:1, v/v) and dissolved in ultrapure water. Then, the metal content was determined by an inductively coupled plasma mass spectrometer (NexION 350X; Shelton, CT, USA) using the GB5009.268-2016 method, in which three biological replicates were set. The National Standard Reference Materials GB W080684 was considered the internal reference to carry out the quality control, and a blank experiment was performed throughout the entire process. The utensils were soaked overnight with 5% nitric acid solution and rinsed with deionized water.

### Detection of Mn Transporter Gene Expression

The total RNA of rice roots was extracted with the Tiangen RNAsimple Total RNA Kit and reverse transcribed into cDNA using the Fermentas quantitative reverse transcription kit. The real-time quantitative PCR system was prepared according to the requirements of TaKaRa’s SYBR Premix EX Taq Quantitation Kit and conducted using the Roche Light Cycler 480 quantitative PCR instrument. The following reaction steps were performed: pre-denaturation at 95°C for 30 s, denaturation at 95°C for 5 s, annealing at 57°C for 30 s, and extension at 72°C for 30 s, with a total number of 40 cycles. For the melting curve analysis, the following protocol was followed: 5 s at 95°C, 1 min at 60°C, and 0 s at 95°C, after which the temperature dropped to 50°C for 30 s. OsActin was considered an internal reference gene, and the expression of the Mn transporter gene in the root samples was estimated using the 2^−ΔΔCt^ method (Livak and Schmittgen, 2001). The primer sequences are presented in **Supplementary Data 2**, **Table S3**.

### Statistical Analysis

Statistical significance was determined by two-tailed Student’s *t*-test for comparison of two groups. Differences were considered statistically significant when *P* ≤ 0.05. *P*-values are indicated by * when *P* < 0.05 or ** when *P* < 0.01 (Zhao et al., 2018).

## Results and Analysis

### *OsNramp5* Sequence Analysis and Selection of Gene-Editing Target Sites

**Figure 1:**
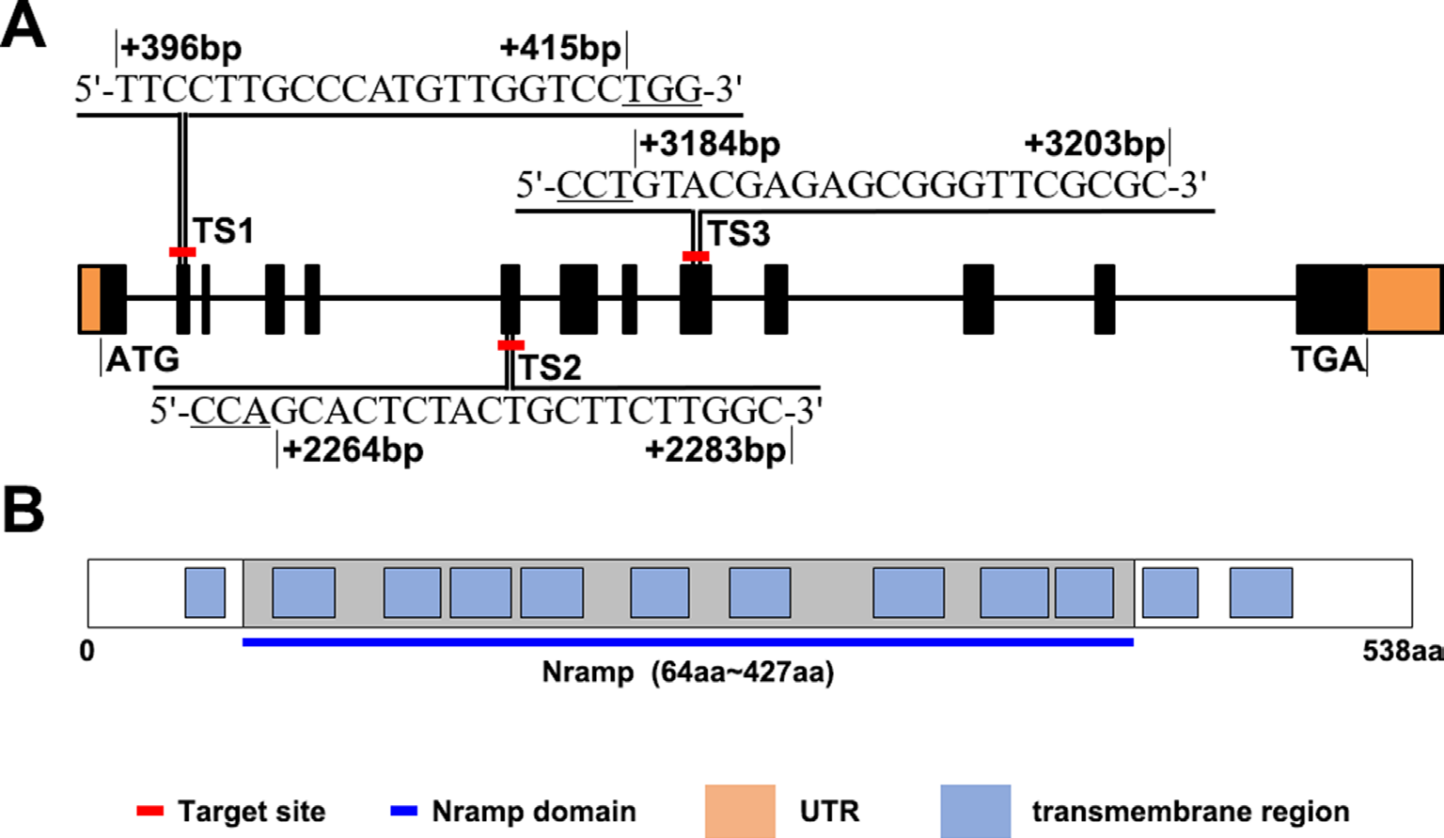
*OsNramp5* sequence and protein sequence patterns. **(A)** *OsNramp5* gene structure pattern, where the orange areas refer to untranslated regions (UTR), the black areas refer to exons, and the black lines refer to introns. The red-labeled TS1, TS2, and TS3 are the gene positions and nucleotide sites of the three target sites, respectively. **(B)** OsNramp5 protein structure pattern, where the blue boxes refer to the transmembrane domains, and the gray areas refer to the Nramp domains. TS1, target site 1; TS2, target site 2; TS3, target site 3.

*OsNramp5* has a full length of 7.47-kbp, distributed in 13 exons, and encodes a transporter containing 538 amino acids (**Figure 1A**). The SMART database was used to analyze the OsNramp5 protein sequence. The results obtained (**Figure 1B**) revealed that OsNramp5 contained 12 transmembrane domains, 9 of which constituted a conserved Nramp domain, which indicates that OsNramp5 belongs to the typical Nramp membrane protein family and plays an important regulatory role in the transport of metal ions (Tabuchi et al., 1999; Thomine et al., 2000; Nevo and Nelson, 2006; Ueki et al., 2011; Mani and Sankaranarayanan, 2018). To explore the effects of different *OsNramp5* mutation sites on the metal ion transport and growth in rice, three CRISPR/Cas9 gene-editing sites were selected. TS1 is located at the second exon (396–415 bp), with a sequence of TTCCTTGCCCATGTTGGTCC; TS2 is located at the sixth exon 2,264–2,283 bp, that is, the front part of the Nramp domain, with a sequence of GCACTCTACTGCTTCTTGGC; and TS3 is located at the ninth exon (3,184–3,203 bp), that is, the middle part of the Nramp domain, with a sequence of GTACGAGAGCGGGTTCGCGC. Subsequently, Basic Local Alignment Search Tool (BLAST) was employed to perform genome-wide alignment of three target sequences, which revealed that TS1, TS2, and TS3 motifs were specific.

### Acquisition of *OsNramp5* Mutants and Mutation-Type Analysis

**Figure 2:**
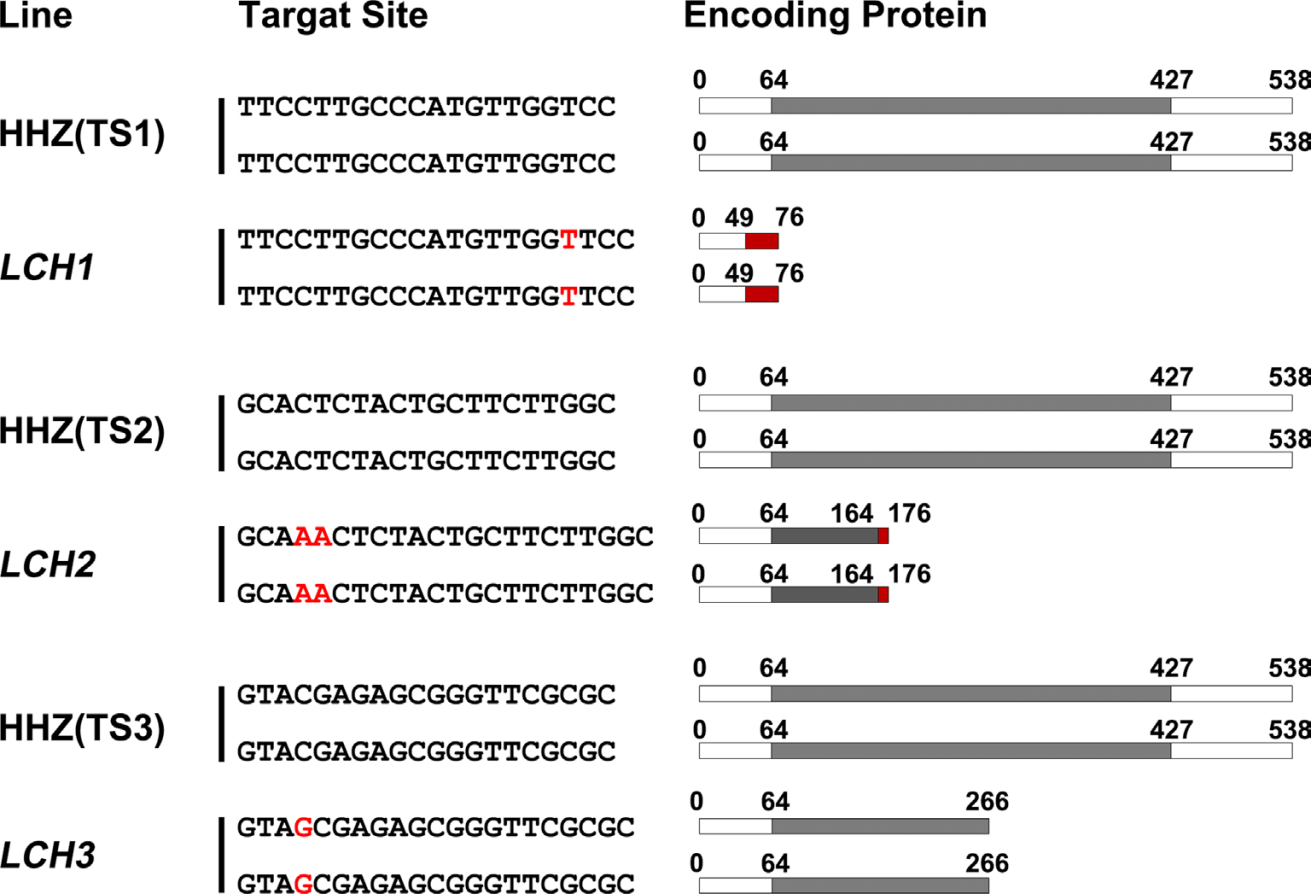
*OsNramp5* mutation types and protein sequences. HHZ (TS1), HHZ (TS2), and HHZ (TS3) are the three target site sequences in *OsNramp5*. *LCH1*, *LCH2*, and *LCH3* are three different types of mutant and mutation site sequences, respectively. The red letters represent the inserted base; the gray areas refer to the Nramp domains; and the red areas denote the new amino acid sequences encoded after the mutation. TS1, target site 1; TS2, target site 2; TS3, target site 3.

The CRISPR/Cas9 plant expression vectors of TS1, TS2, and TS3 were constructed, and the *O. sativa* subsp. *indica* variety Huanghuazhan was transformed to obtain transgenic plants. Then T_0_ generation transformed plants were preferably screened by sequencing and then labeled as *LCH1*, *LCH2*, and *LCH3* (the mutation types are presented in **Figure 2**). For *LCH1*, a T was inserted between the third and fourth bases before the protospacer adjacent motif (PAM) of TS1, and a frameshift mutation was encoded at the 49th amino acid and terminated in advance at the 76th amino acid, which resulted in complete deletion of the Nramp domain. For *LCH2*, an AA was inserted between the third and fourth bases before the PAM of TS2, and a frameshift mutation was encoded at the 164th amino acid, which was terminated in advance at the 176th amino acid, which encoded a Nramp domain with 100 amino acid residues, leading to partial deletion of the Nramp domain. For *LCH3*, a G was inserted between the third and fourth bases before the PAM of TS3, which encoded the 266th amino acid encoding a Nramp domain with 202 amino acid residues that resulted in partial deletion of the Nramp domain. Therefore, *LCH1*, *LCH2*, and *LCH3* are functional deletion mutants of *OsNramp5*. However, due to the different mutation sites and types, *LCH1*, *LCH2*, and *LCH3* are different in the degree of *OsNramp5* gene function deletion. Hence, the effects of different mutation types of *OsNramp5* on important agronomic traits of rice are worthwhile for further studies.

### Different Mutation Types of *OsNramp5* Affect the Accumulation of Metal Ions in Rice Grains

**Figure 3:**
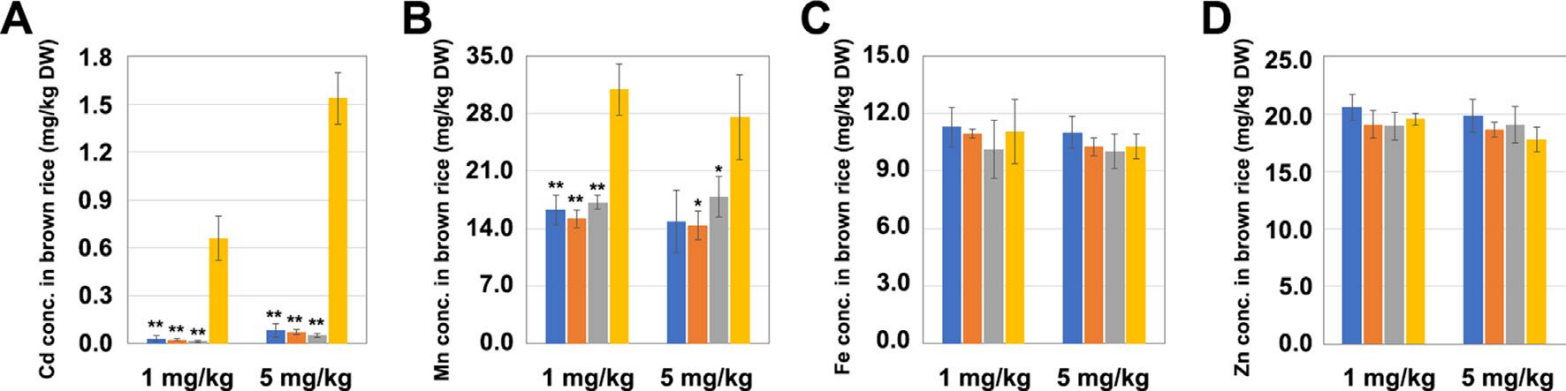
Effects of different mutation types of *OsNramp5* on the accumulation of metal ions in rice grains. The measured values of the grain metal accumulation in *LCH1*, *LCH2*, *LCH3*, and WT, planted in the soil treated with Cd concentrations of 1 and 5 mg/kg. **(A)** Cd content in grains. **(B)** Mn content in grains. **(C)** Fe content in grains. **(D)** Zn content in grains. The blue columns represent *LCH1*; the orange columns represent *LCH2*; the gray columns represent *LCH3*; the yellow columns represent control. Data are given as means ± SD, with three biological replicates. * or ** significant difference (*P* < 0.05 or 0.01, respectively, *t*-test). WT, wild type

The T_1_ generation *LCH1*, *LCH2*, and *LCH3* homozygous mutants were planted in pots with soil that was then treated in three replicates with different Cd concentrations (1 and 5 mg/kg), in which the Mn concentration was set to 350 mg/kg. In the soil treated with a Cd concentration of 1 mg/kg, the Cd contents in the grains of *LCH1*, *LCH2*, and *LCH3* were exceedingly low (0.032, 0.025, and 0.016 mg/kg, respectively), which were decreased by 95%, 96%, and 97%, correspondingly, compared with those of 0.659 mg/kg in the control group, and were lower than the upper limit of Chinese National Standard for Cd content of 0.2 mg/kg (**Figure 3A**). Moreover, the Mn concentrations in the grains of *LCH1*, *LCH2*, and *LCH3* were also lower than those of the control group after the treatment with a Cd concentration of 1 mg/kg (**Figure 3B**). Meanwhile, no significant differences were observed in the Fe and Zn contents of the grains of *LCH1*, *LCH2*, and *LCH3* after the treatment with a Cd concentration of 1 mg/kg compared with those of the control (**Figures 3C, D**). The accumulation of metals in *LCH1*, *LCH2*, and *LCH3* grains planted in the soil after the treatment with a Cd concentration of 5 mg/kg is also shown in **Figures 3A–D**, which was consistent with that in soil after treatment with Cd of 1 mg/kg concentration. It is inferred that the different types of mutants of *OsNramp5* are not sensitive to the change of soil Cd concentration, and the functional deficiency of *OsNramp5* can reduce the contents of Cd and Mn in grains, in which the Cd and Mn show the same accumulation trend.

### Different Mutation Types of *OsNramp5* Affect Rice Yield Traits

**Figure 4:**
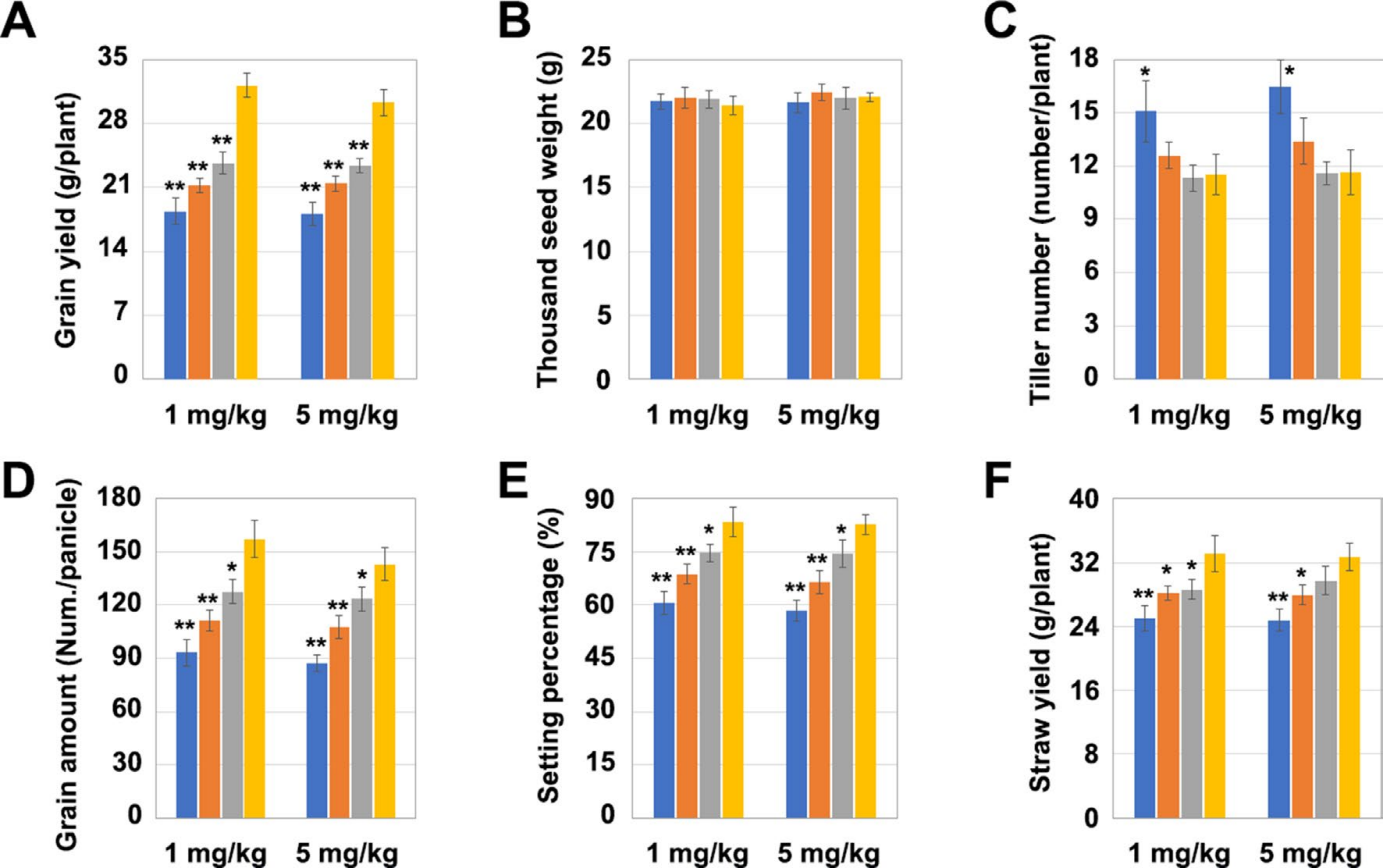
Effects of different mutation types of *OsNramp5* on rice yield. **(A)**–**(F)** Statistical analysis of the yield per plant, 1,000 grain weight, number of tillers, grain number per ear, seed-setting rate, and straw yield of *OsNramp5* mutants *LCH1*, *LCH2*, and *LCH3* and control after treatment with Cd of 1 and 5 mg/kg concentrations. The blue columns represent *LCH1*; the orange columns represent *LCH2*; the gray columns represent *LCH3*; the yellow columns represent control. Data are given as means ± SD, with three biological replicates. * or ** significant difference (*P* < 0.05 or 0.01, respectively, *t*-test)

The yield per plant of *LCH1*, *LCH2*, and *LCH3* was significantly lower than that of the control after the treatment with a Cd concentration of 1 mg/kg but increased with the decrease of the *OsNramp5* mutation degree (**Figure 4A**). Subsequently, the four key indicators affecting the yield per plant (1,000 grain weight, number of tillers, grain number per ear, and seed-setting rate) were further analyzed. The results revealed that the 1,000 grain weight of *LCH1*, *LCH2*, and *LCH3* was not significantly different from that of the control (**Figure 4B**). Nevertheless, the number of tillers of *LCH1* was significantly higher than that of the control, whereas the number of the tillers of *LCH2* and *LCH3* were not significantly changed compared with that of the control. This result indicates that the number of tillers rises with the increase in the degree of the *OsNramp5* mutation (**Figure 4C**). The grain number per ear and seed-setting rate of *LCH1*, *LCH2*, and *LCH3* were significantly lower than those of the control, but the decline in the degree of *OsNramp5* mutation considerably increased them (**Figures 4D, E**). In addition, *LCH1*, *LCH2*, and *LCH3* showed a significant lower straw yield than that of the control but exhibited also a trend of increasing the yield as the degree of *OsNramp5* mutation was weakened (**Figure 4F**). With the treatment with Cd of 5 mg/kg concentration, the agronomic traits of *LCH1*, *LCH2*, and *LCH3* were similar to those after treatment with Cd of 1 mg/kg concentration (**Figures 4A–F**).

### Effects of Different Mutation Types of *OsNramp5* on Grain Quality

**Figure 5:**
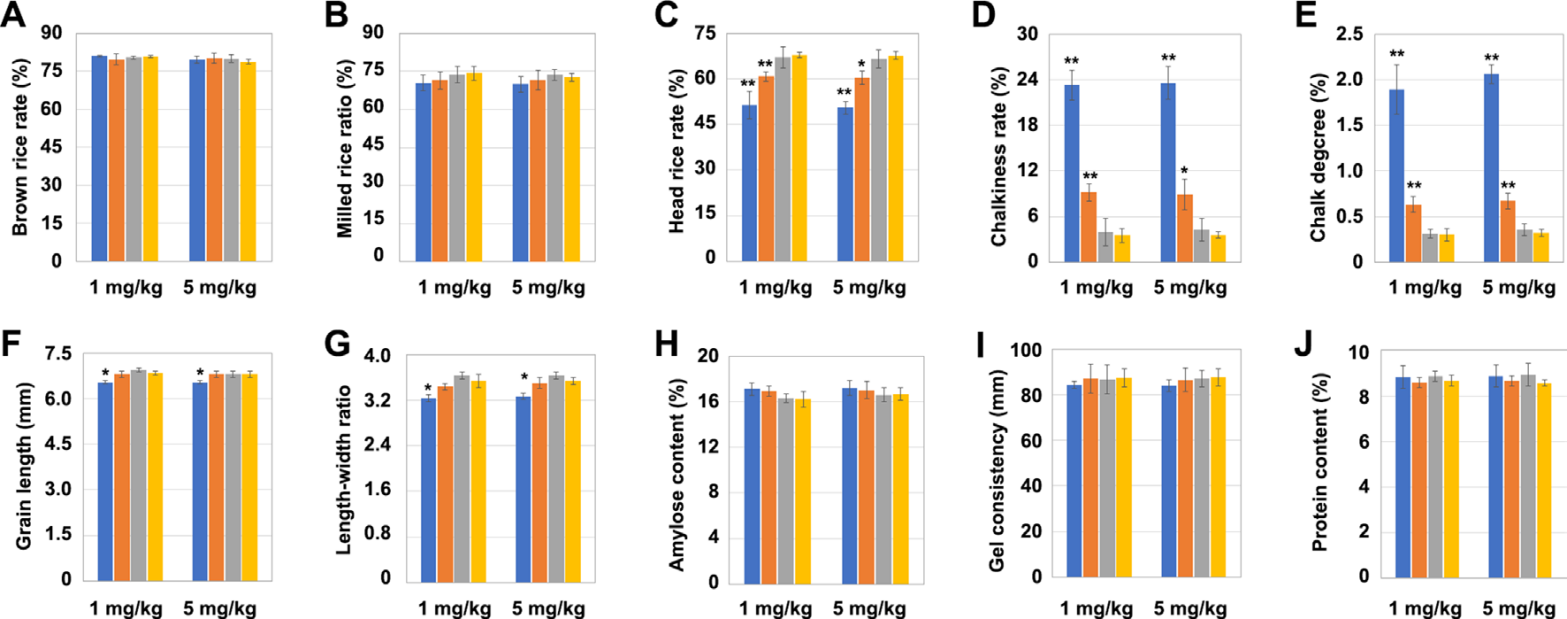
Effects of different mutation types of *OsNramp5* on grain quality traits. **(A)**–**(J)** Statistics on brown rice rate, milled rice rate, head rice rate, chalky grain rate, chalkiness degree, grain length, aspect ratio of grains, amylose content, gel consistency, and protein content of *OsNramp5* mutants *LCH1*, *LCH2*, and *LCH3* and control after treatment with Cd concentrations of 1 and 5 mg/kg. The blue columns represent *LCH1*; the orange columns represent *LCH2*; the gray columns represent *LCH3*; the yellow columns represent control. Data are given as means ± SD, with three biological replicates. * or ** significant difference (*P* < 0.05 or 0.01, respectively, *t*-test)

The grains of the aforementioned mutants were comprehensively evaluated in terms of their quality characteristics: processing, appearance, eating, and nutritional qualities. The *OsNramp5* mutation had no significant effect on the brown rice rate and milled rice rate in the grains (**Figures 5A, B**). But the head rice rate was significantly lower in the *LCH1* and *LCH2* groups than that in the control but increased along with the degree of *OsNramp5* mutation reduction, although no significant difference existed between the *LCH3* and the control groups (**Figure 5C**). The chalkiness degree and chalky grain rate were significantly higher in the *LCH1* and *LCH2* groups than in the control group but significantly declined as the degree of *OsNramp5* mutation was weakened and did not show a significant difference between the *LCH3* and the control group (**Figures 5D, E**). In addition, the *OsNramp5* mutation affected also the length and aspect ratio of grains (**Figures 5F, G**) but had no significant effect on the amylose content, gel consistency, and protein content in the grains (**Figures 5H, I, J**). Furthermore, the above quality indicators had a trend consistent with the post-treatment effects of Cd concentrations of 1 and 5 mg/kg. Therefore, the mutation of *OsNramp5* exerts significant negative effects on the chalkiness degree and chalky grain rate, but these quality traits were alleviated by changing the mutation site of *OsNramp5*.

### Effects of Different Mutation Types of *OsNramp5* on the Accumulation of Mn in the Chlorenchyma

**Figure 6:**
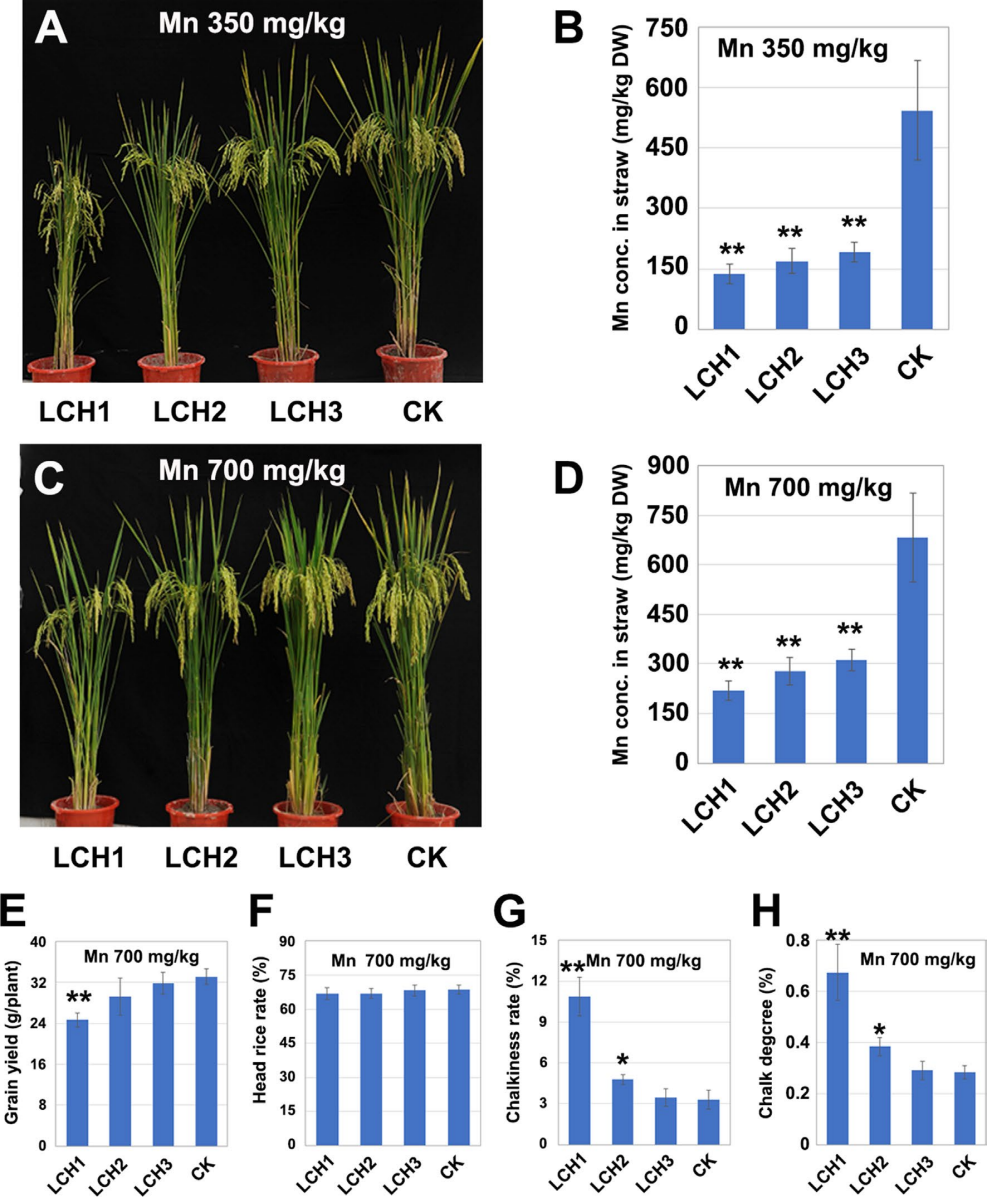
Relationship between Mn concentration in the chlorenchyma of mutants and the yield and quality of rice. **(A)** Phenotype of *LCH1*, *LCH2*, *LCH3*, and control with a soil Mn content of 350 mg/kg. **(B)** Mn content in chlorenchyma of *LCH1*, *LCH2*, *LCH3*, and control at maturity with a soil Mn content of 350 mg/kg. **(C)** Phenotype of *LCH1*, *LCH2*, *LCH3*, and control with a soil Mn content of 700 mg/kg. **(D)** Mn content in chlorenchyma of *LCH1*, *LCH2*, *LCH3*, and control at maturity with a soil Mn content of 700 mg/kg. **(E)** Yield of *LCH1*, *LCH2*, *LCH3*, and control with a soil Mn content of 700 mg/kg. **(F)** Milled rice rate of *LCH1*, *LCH2*, *LCH3*, and control with a soil Mn content of 700 mg/kg. **(G)** Chalky grain rate of *LCH1*, *LCH2*, *LCH3*, and control with a soil Mn content of 700 mg/kg. **(H)** Chalkiness degree of *LCH1*, *LCH2*, *LCH3*, and control with a soil Mn content of 700 mg/kg. Data are given as means ± SD, with three biological replicates. * or ** significant difference (*P* < 0.05 or 0.01, respectively, *t*-test)

The T_1_ generation of *LCH1*, *LCH2*, and *LCH3* homozygous mutants was planted in pots with that was then treated in three replicates with different Mn concentrations (350 and 500 mg/kg), in which the Cd concentration was set to 1mg/kg. The results showed that the Mn contents in the chlorenchyma of *LCH1*, *LCH2*, and *LCH3* at maturity were lower than those of the control but increased with the decrease of the *OsNramp5* mutation degree. Meanwhile, the Mn contents in the chlorenchyma of *LCH1*, *LCH2*, and *LCH3* increased with the elevation of the soil Mn concentration (**Figures 6B, D**). In addition, the yield per plant rose in the *LCH1*, *LCH2*, and *LCH3* groups with the increase of the chlorenchyma Mn concentration, when the soil was treated with 700 mg/kg Mn, as compared with the treatment with 350 mg/kg Mn, where the yield per plant of the *LCH3* returned to the level of the control (**Figures 4A** **and** **6A, C, E**). The head rice rate increased in the *LCH1* and *LCH2* groups with the elevation of the chlorenchyma Mn concentration, which was basically restored to the level of the control; no significant difference was detected between the *LCH3* and the control (**Figures 5C** **and** **6F**). With the increase of chlorenchyma Mn concentration, the chalkiness degree and chalky grain rate were decreased in *LCH1*, *LCH2*, and *LCH3* groups at various degrees, of which those in the *LCH2* group were closer to the levels in the control; no significant difference was detected between the *LCH3* and the control groups (**Figures 5D, E** **and** **6G, H**).

### Effects of Different Mutation Types of *OsNramp5* on Mn Transporter Expression

**Figure 7:**
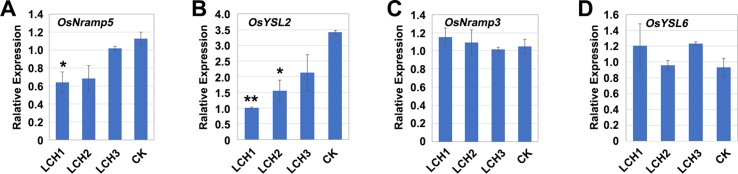
Expression of Mn transporter genes in the roots of different *OsNramp5* mutants. **(A)**–**(D)** Statistical data of the expression of *OsNramp5*, *OsYSL2*, *OsNramp3*, and *OsYSL6* of *OsNramp5* mutants *LCH1*, *LCH2*, *LCH3*, and control. Data are given as means ± SD, with two biological replicates. * or ** significant difference (*P* < 0.05 or 0.01, respectively, *t*-test)

The transporters encoded by *OsNramp5*, *OsYSL2*, *OsNramp3*, and *OsYSL6* are critically involved in the absorption and transport of Mn in rice (Ishimaru et al., 2010; Sasaki et al., 2011; Yamaji et al., 2013). When a plant lacks a necessary nutritious element, it will induce upregulation of the relevant transporter gene to promote the absorption and transport of the element (Ishimaru 2005; Ishimaru et al., 2007; Takahashi et al., 2011). During the tillering period of rice, the expressions of *OsNramp5* and *OsYSL2* in the mutants *LCH1*, *LCH2*, and *LCH3*, as compared with the control, were downregulated to varying degrees, whereas the expression levels of *OsNramp3* and *OsYSL6* were not significantly changed (**Figures 7A–D**). Therefore, the functional deficiency of *OsNramp5* leads to the downregulation of *OsYSL2*, and *OsYSL2* is a key gene for Mn absorption in rice roots, which indicates that the functional deficiency of *OsNramp5* affects simultaneously the absorption and transport of Cd and Mn. Our further analysis revealed that gradual upregulation of *OsYSL2* expression occurred with the decrease of *OsNramp5* mutation in *LCH1*, *LCH2*, and *LCH3* mutants (**Figures 7A, B**). Thus, the Mn absorption pathway was restored to a certain extent, which alleviated the effect of Mn ion on rice growth and development.

## Discussion

*OsNramp5* is a key gene for controlling the uptake of Cd, Mn, and other metal ions by rice root cells. The functional deficiency of this gene can significantly reduce the accumulation of Cd in rice grains, but the effects of its mutation on agronomic traits such as yield and quality have not been reported comprehensively yet. To determine the effects of a mutation at different target sites of *OsNramp5* on grain Cd accumulation and other important agronomic traits of rice, we obtained three Huanghuazhan-based *OsNramp5* mutants *LCH1*, *LCH2*, and *LCH3* using CRISPR/Cas9 gene-editing technology. The results of the statistical analysis of Cd accumulation and certain other important agronomic traits of grains of the above three mutants revealed that the Cd accumulation in *LCH1*, *LCH2*, and *LCH3* grains was extremely low. *OsNramp5* mutation reduced also the yield per plant and the head rice rate but increased the chalkiness degree and chalky grain rate. Nonetheless, with the decrease of the *OsNramp5* mutation degree, the yield and quality of grains were restored to some extent.

Mn is a mineral element indispensable for rice growth and development, which mainly acts as a cofactor for photosynthesis-related enzymes and participates in the oxygen evolution of photoreaction in rice cells. A deficiency of Mn leads to blockage of the photosynthesis in rice, eventually resulting in a decline in the yield (Alejandro et al., 2017; Farzadfar et al., 2017; Bloom and Lancaster, 2018). Previous studies showed that the Mn content in the chlorenchyma of *OsNramp5* mutants was significantly lower than that of the WT. The insufficient absorption of Mn affected the development of the mutants, whereas the high external concentration of Mn gradually restored the phenotype of the *OsNramp5* mutants. Recent investigations revealed that the DPGN motif of the first transmembrane domain of *OsNramp5* influenced the transport of Mn (Cellier et al., 1995; Lam-Yuk-Tseung et al., 2003; Cohen et al., 2003; Haemig and Brooker, 2004; Czachorowski et al., 2009). In addition, after the insertion of the transposon into the 10th exon of *OsNramp5*, the encoded protein residues were still anchored on the cell membrane, exerting transport Mn (Sasaki et al., 2012). To explore the effects of different mutation types of *OsNramp5* on yield, we investigated the accumulation of Mn and the expression of Mn transporter genes. Our results showed that the concentration of Mn in the chlorenchyma increased in the *OsNramp5* mutants *LCH1*, *LCH2*, and *LCH3* with the decrease in the mutation degree; the yield per plant also rose. In addition, the treatment with a Mn concentration of 700 mg/kg restored the yield per plant of *LCH3* to the level of the control. Meanwhile, the chalky grain rate and chalkiness degree decreased to a varying extent with the elevation of the Mn concentration in the chlorenchyma of *LCH1*, *LCH2*, and *LCH3*. In a future study, we intend to backcross the mutants with the original variety Huanghuazhan to further elucidate the mechanisms through which the *OsNramp5* mutation affects rice quality. Additionally, investigations of the mutations of other exons of *OsNramp5* are also required to establish the most suitable mutation site, so that the mutants can achieve the WT phenotype in a soil with a low Mn concentration.

In the present study, the Cd content in the grains of the mutants was extremely low; these mutants had a great potential application in soils contaminated with Cd pollution. Their practical application may be restricted due to the off-target phenomenon of the CRISPR/Cas9 gene-editing technology (Duan et al., 2014; Shen et al., 2014; Kim et al., 2015; Kleinstiver et al., 2016). A previous study suggested that CRISPR/Cas9 editing specificity is only related to 7- to 12-bp bases before PAM (Pattanayak et al., 2013). We found 333, 186, and 231 sequences in the rice genome-wide alignment with the top 8 bp of the PAM of the target sites TS1, TS2, and TS3, respectively. We further performed genomic resequencing of *LCH1*, *LCH2*, and *LCH3* and aligned the above matched sequences one by one. Next, we found that *LCH1*, *LCH2*, and *LCH3* had no single-nucleotide polymorphism (SNP) or indel in other corresponding regions except for the mutations at the target sites of *OsNramp5* (**Supplementary Table S1**, **Figure S1**). In addition, we aligned the CRISPR/Cas9 plant binary expression vector sequence with the resequenced sequence and established that *LCH1*, *LCH2*, and *LCH3* did not contain transgenic components and could be directly applied to low-cadmium rice breeding.

Furthermore, the rice *OsNramp5* gene sequences were aligned with Rice Functional Genome Breeding Database (RFGB), which included SNP and insertion/deletion (InDel) of 3,000 rice (3K rice) genomes worldwide (Zhang et al., 2015). A total of 35 SNPs, and 1 InDel were found in the coding sequence of *OsNramp5*. Based on the *OsNramp5* mutation site and type, a total number of 216 rice candidate varieties were obtained, which might have been low Cd accumulating (the sites and materials used are listed in **Supplementary Data 3**). In summary, the mutation screening of *OsNramp5* 3K rice can effectively guide the selection of new low-Cd-accumulating rice varieties with excellent agronomic traits and adaptability. This study provides novel insights into and new materials for breeding new rice varieties with low Cd accumulation and good agronomic traits under conditions of severe Cd pollution.

## Data Availabilty

All datasets for this study are included in the manuscript and the supplementary files.

## Author Contributions

LL and JLW designed the research work and drafted the manuscript. TW performed the experiments, analyzed the data, and drafted the manuscript. YL and DY modified the draft. YF, HX, SS, MQ, JW, MC, GC, YT, and CL participated in performing the experiments.

## Funding

This work was supported by the Major Scientific Research Project of Hunan Province (Grant No. XCNZ [2018] 83), by the National Natural Science Foundation of China (31671669), by Ministry of Science and Technology of the People’s Republic of China (2017YFD0100400), and by Hunan Provincial Science and Technology Department (2016NK2204). The CRISPR/Cas9 editing vectors, *pYLgRNA-U3*, and *pYLCRISPR/Cas9-MH* were provided by Prof. Yaoguang Liu, South China Agricultural University.

## Conflict of Interest Statement

The authors declare that the research was conducted in the absence of any commercial or financial relationships that could be construed as a potential conflict of interest.
