# Supplementary material for: Mutation at Different Sites of Metal Transporter Gene OsNramp5 Affects Cd Accumulation and Related Agronomic Traits in Rice (Oryza sativa L.)
Source: Front Plant Sci. 2019 Sep 11;10:1081. doi: 10.3389/fpls.2019.01081 (PMC6749076; doi:10.3389/fpls.2019.01081)
Supplement: Supplementary file 1 [file Table_1.docx]

**Supplementary data 1：Genomic editing sites and insertional mutation pattern of OsNramp5 mutants**

**Table S1. Detection results of potential off-target sites.**

| Target site number | Target sequence | potential off-target sequence | Number of potential off-target sites | Number of off-targets (except target sites) |
| --- | --- | --- | --- | --- |
| TS1 | TTCCTTGCCCATGTTGGTCCTGG | NNNNNNNNNNNNGTTGGTCCNGG | 333 | 0 |
| TS2 | GCCAAGAAGCAGTAGAGTGCTGG | NNNNNNNNNNNNTAGAGTGCNGG | 186 | 0 |
| TS3 | GCGCGAACCCGCTCTCGTACAGG | NNNNNNNNNNNNTCTCGTACNGG | 231 | 0 |


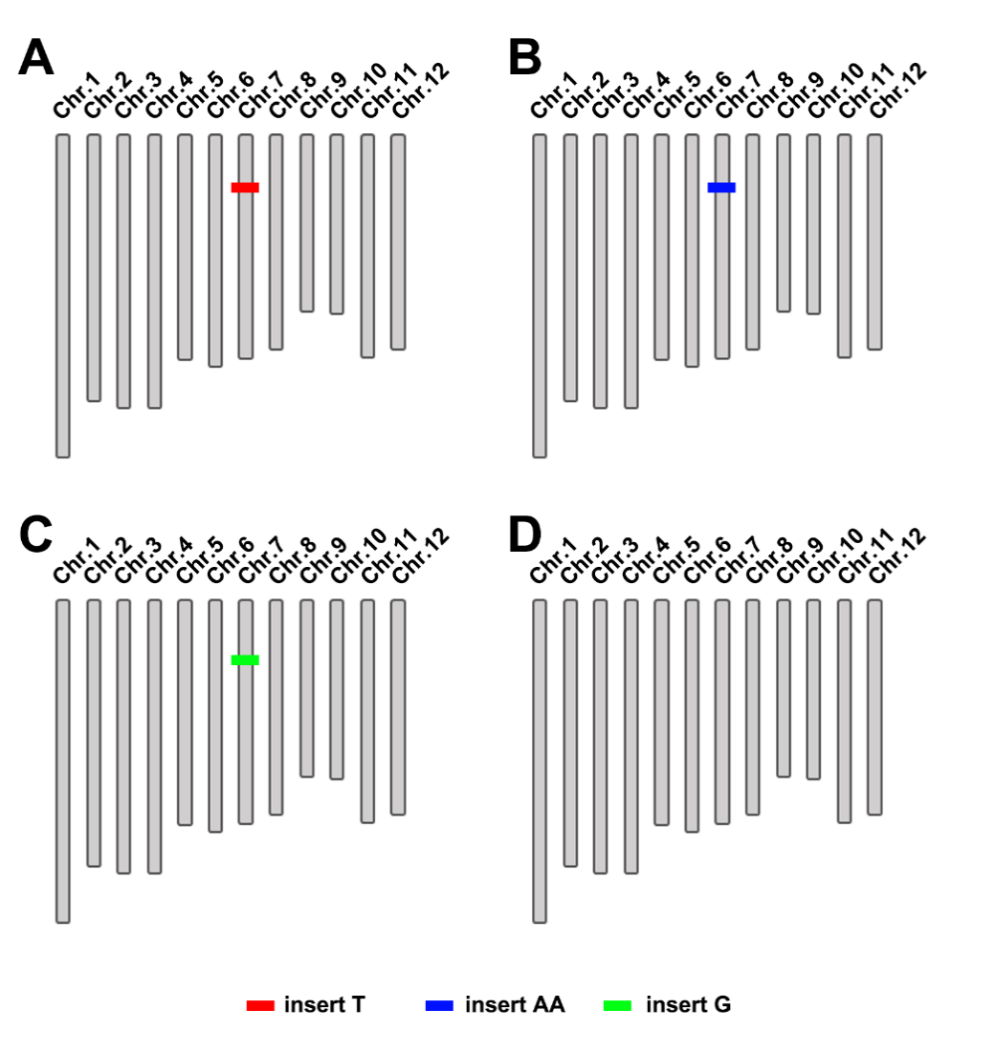


Figure S1. Genomic editing sites and insertional mutation pattern of *OsNramp5* mutants

(A) Mutant *LCH1*: a T base is inserted to the chromosome 7, and no mutation occurs at other sites;

(B) Mutant *LCH2*: a AA base is inserted to the chromosome 7, and no mutation occurs at other sites；

(C) Mutant *LCH3*: a G base is inserted to the chromosome 7, and no mutation occurs at other sites；

(D) Wild-type control
