# Supplementary material for: Mutation at Different Sites of Metal Transporter Gene OsNramp5 Affects Cd Accumulation and Related Agronomic Traits in Rice (Oryza sativa L.)
Source: Front Plant Sci. 2019 Sep 11;10:1081. doi: 10.3389/fpls.2019.01081 (PMC6749076; doi:10.3389/fpls.2019.01081)
Supplement: Supplementary file 2 [file Table_2.docx]

**Supplementary data 2：Primer name and primer sequences**

Table S2. Primer and sequences

| primer name | sequence of forward primer(5′-3′) | sequence of reverse primer(5′-3′) |
| --- | --- | --- |
| SP1/SP2 | CCCGACATAGATGCAATAACTTC | GCGCGGTGTCATCTATGTTACT |
| primer-*LCH1* | CTTTCAGTCATTCAGTGCGTAA | GCATGGCATTCATTGCTC |
| primer-*LCH2* | ACTTGACAATCGATCCAACTAGC | CGAAGCTTTGCTGATCGGG |
| primer-*LCH3* | AGGAAGGAGGAGGTGTCGAG | CTATCGAGGAAGACACCGGC |

SP1/SP2: primer for detecting positive plants，primer-*LCH1*: Amplification primers for detecting mutations in the TS1 target site，primer-*LCH2*: Amplification primers for detecting mutations in the TS2 target site，primer-*LCH3*: Amplification primers for detecting mutations in the TS3 target site

Table S3. Internal reference and Mn transporter gene primer sequence

| gene | sequence of forward primer(5′-3′) | sequence of reverse primer(5′-3′) |
| --- | --- | --- |
| *OsActin* | CAGGCCGTCCTCTCTCTGTA | AAGGATAGCATGGGGGAGAG |
| *OsNramp5* | TTCGTTTATATTTGTGCGGTCC | CACCTCCCCTCAAATGCTTATA |
| *OsYSL2* | AGCGACAGATGAGGATACATTT | GTTGGAACATTATGGGTATCGC |
| *OsNramp3* | CAATTACAGGAACTTATGCGGG | TTGCCAAGGACCTAGTTAGAAG |
| *OsYSL6* | GCAATCATATCTTGGGGTTTCC | CCTTGTATCCGTATAGCCCTTT |
